# Comparing the effectiveness of the 0.018-inch versus the 0.022-inch bracket slot system in orthodontic treatment: study protocol for a randomized controlled trial

**DOI:** 10.1186/1745-6215-15-389

**Published:** 2014-10-06

**Authors:** Ahmed MF El-Angbawi, David R Bearn, Grant T McIntyre

**Affiliations:** Dundee Dental Hospital & School, Park Place, University of Dundee, Dundee, DD1 4HN UK

**Keywords:** Orthodontic treatment, Bracket slot size, Effectiveness, Randomized clinical trial, Clinical outcomes, Duration of treatment

## Abstract

**Background:**

Edgewise fixed orthodontic appliances are available in two different bracket slot sizes (0.018 and 0.022 inch). Both systems are used by clinicians worldwide with some orthodontists claiming the superiority and clinical advantages of one system over the other. However, the scientific evidence supporting this area is scarce and weak. This leaves the clinician’s choice of bracket slot system to clinical preference. We aim to compare the 0.018-inch and 0.022-inch pre-adjusted bracket slot systems in terms of the effectiveness of orthodontic treatment.

**Methods/Design:**

This is a prospective, multicenter, randomized clinical trial, undertaken in the secondary care hospital environment in the NHS Tayside region of Scotland (United Kingdom). A total of 216 orthodontic patients will be recruited in three centers in secondary care hospitals in NHS Tayside. The participants will be randomly allocated to treatment with either the 0.018-inch or 0.022-inch bracket slot systems (n = 108 for each group) using Victory series™ conventional pre-adjusted bracket systems (3 M Unitek, Monrovia, United States). Baseline records and outcome data collected during and at the end of orthodontic treatment will be assessed. The primary outcome measures will be the duration of orthodontic treatment in the maxillary and mandibular arches. The secondary outcome measures will be the number of scheduled appointments for orthodontic treatment in the maxillary and mandibular arches, treatment outcome using Peer Assessment Rating index (PAR), orthodontically induced inflammatory root resorption (as measured using periapical radiographs) and the patient’s perception of wearing orthodontic appliances.

**Discussion:**

The results from the current study will serve as evidence to guide the clinician in deciding whether the difference in bracket slot size has a significant impact on the effectiveness of orthodontic treatment.

**Trial registration:**

Registered with ClinicalTrials.gov on 5 March 2014, registration number: NCT02080338.

## Background

Malocclusion is a common worldwide dental problem, often causing aesthetic and functional concerns, which can lead to a negative impact on the quality of life [1, 2]. Malocclusion is normally corrected using orthodontic treatment which includes fixed and/or removable braces. There are a number of fixed appliance systems used in contemporary orthodontics which include multiple pre-adjusted edgewise fixed appliance systems. Edward H Angle is regarded as the father of modern orthodontics and introduced the ‘edgewise’ bracket into orthodontic treatment in 1925 as a development of the ‘ribbon arch’ appliance. The interaction between the edgewise bracket slot and the orthodontic archwire can determine the nature of the orthodontic forces. In order that the gold archwires were sufficiently rigid, Angle proposed the bracket slot dimensions be 0.022 by 0.028 inches. The advent of stainless steel alloys facilitated the use of smaller dimension wires with the same rigidity as that of the larger gold alloy archwires. This allowed the bracket slot size to be reduced, and as a result the 0.018-inch bracket slot was introduced into orthodontics. However, the introduction of the 0.018-inch bracket slot did not eliminate fixed appliance systems using 0.022-inch bracket slots from clinical practice [3].

In the last 20 years, a number of other alloy archwires have been developed. These include standard nickel titanium, thermally activated nickel titanium, superelastic nickel titanium and titanium-molybdenum archwires. Despite these advances in metallurgy, no high quality studies have sought to investigate the effectiveness of 0.018-inch compared with 0.022-inch bracket slots in orthodontic treatment. Only two retrospective studies have compared the duration of treatment for patients treated with 0.018-inch slot and 0.022-inch slot fixed appliance systems [4, 5]. It is interesting to note that these two studies reported a statistically significant reduction in the mean orthodontic treatment duration for the 0.018-inch slot system; however, this difference was not considered by the authors to be clinically significant.

A number of biomechanical advantages and disadvantages have been suggested for both 0.018-inch and 0.022-inch bracket slots. It has been postulated that overbite reduction (a key stage in fixed appliance orthodontic treatment) and closure of any residual extraction space may be more efficient with 0.022-inch bracket slots as the space between the working archwire (0.019 by 0.025-inch stainless steel) and the 0.022-inch bracket slot (known as slop) allows exaggerated bite-opening bends to be placed whilst still being able to be fitted with relative ease [6]. Conversely, the working archwire (0.016 by 0.022-inch stainless steel) for 0.018-inch bracket slots is presumed to deliver third order movement (known as torque) more effectively and earlier in treatment without additional wire bending [6].

All fixed appliance orthodontic treatment results in adverse effects on the roots of the teeth, the most common being orthodontically induced inflammatory root resorption (OIIRR). Excessive forces during treatment have been associated with iatrogenic damage including OIIRR, periodontal destruction, loss of crestal bone height and loss of pulp vitality. It is not known if these are actually significantly reduced when 0.018-inch bracket slots are used, although different appliances are associated with differing levels of biological trauma during treatment [7]. A retrospective study by Sameshima and Sinclair [8] which investigated multiple treatment factors for the prediction and prevention of OIIRR reported that difference in bracket slot size was not significantly associated with OIIRR.

The preference for each slot size varies throughout the world. In the United Kingdom, the majority of orthodontists continue to use 0.022-inch bracket slots. However, in the rest of mainland Europe, 0.018-inch bracket slots are more commonly used, whilst in the United States, the majority of orthodontists work with 0.022-inch bracket slots. It has been estimated that approximately 10% of Americans relocate to another state each year and this is likely to also be true for Europe and the United Kingdom [9]. When patients undergoing orthodontic treatment transfer to a different orthodontist, the dichotomy in orthodontic bracket sizes is not only inconvenient for the new orthodontist if appropriate inventory is not kept in stock, but unnecessary potential side-effects can occur if active treatment is prolonged. These biological and clinical management problems could all be avoided or minimized if one slot size was shown to be superior and subsequently adopted by the profession as the international standard [3, 9].

The aim of this trial is to provide reliable evidence for clinicians as to whether the use of 0.018-inch or 0.022-inch bracket slot systems has any impact on the effectiveness of orthodontic treatment. We will do this by determining if the use of 0.018-inch and 0.022-inch bracket slot systems has any effect on the duration or quality of result of orthodontic treatment, patient’s perception of treatment or the biological side effects associated with orthodontic treatment. We hypothesize that there is no difference in the outcomes of orthodontic treatment (as listed above) using either a 0.018-inch or 0.022-inch slot fixed appliance system.

## Methods/Design

### Trial design

**Figure 1:**
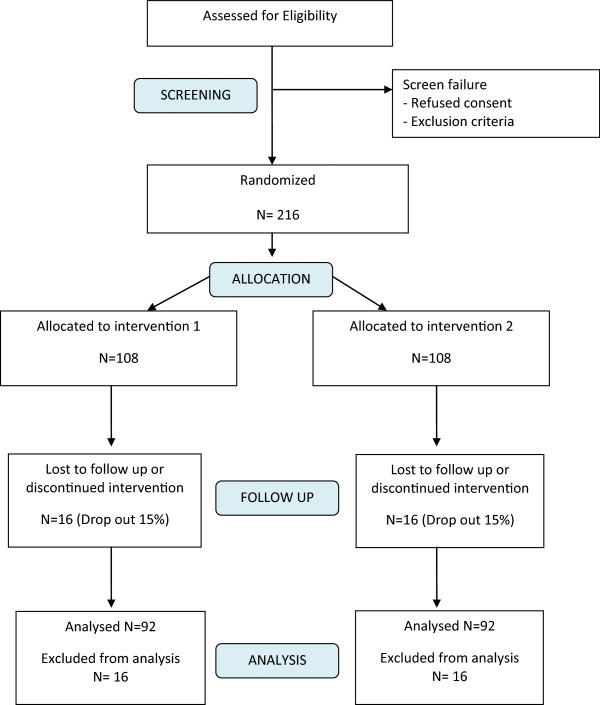
**Trial CONSORT flow chart with projected numbers of participants throughout the trial.** Intervention 1: 0.018-inch bracket slot system and intervention 2: 0.022-inch bracket slot system.

The current trial is a multicenter, two-arm, parallel group, randomized controlled trial (Figure 1) designed following the CONSORT guidelines [10]. Patients who give informed consent to participate in this study will be entered into the study database, which will be held on one of the computers within the Orthodontic Department at Dundee Dental Hospital and School. Each participating patient will be allocated the next available study number at the start of active treatment using a series of opaque envelopes containing the treatment group.

This trial will recruit orthodontic patients from three sites in secondary care setting in NHS Tayside, Scotland: Dundee Dental Hospital and School, Perth Royal Infirmary and Springfield Medical Centre (Arbroath). Prior to study recruitment at any individual site, there will be a site initiation visit and site staff will receive training on the study design, methodology, clinical intervention and maintaining trial documentation, including the case report forms questionnaires and investigator site file.

### Participant inclusion criteria

Patients scheduled to undergo dual arch fixed appliance orthodontic treatment in any of the three trial centers will be invited to participate in this trial by the operator planning to conduct their orthodontic treatment. Patients who are eligible to receive treatment in the NHS Scotland should have a significant need of treatment which requires an Index of Orthodontic Treatment Need (IOTN) score of 4 or 5 for the dental health component, or a score of 3 combined with a high aesthetic component score (>5) [11]. All types of malocclusions (Class I, II and III malocclusions) will be recruited to the study including cases which require extraction or non-extraction as part of the treatment plan.

### Participant exclusion criteria

Participants will be excluded if they fulfil any of the following criteria: patients who have undergone previous orthodontic treatment including fixed, removable and functional appliances; patients less than 12-years-old at the start of orthodontic treatment; patients with orofacial clefting, severe hypodontia and special needs patients; and patients undergoing orthognathic (jaw) surgery as part of their orthodontic treatment plan.

### Intervention protocol

All teeth will undergo pumice and water prophylaxis immediately before bonding and banding. The teeth will then be prepared using self-etching primer (Transbond™ Plus Self Etching Primer, 3 M Unitek, Monrovia, United States). Depending on the card inside the participant’s envelope, either 0.018-inch or 0.022-inch slot Victory Series™ McLaughlin, Bennett, Terevisi (MBT) prescription adhesive pre-coated (APC) brackets and buccal tubes (or bands, where appropriate) (APC™ II Victory Series Twin MBT™, 3 M Unitek, Monrovia, United States] will be placed. A standardized wire sequence for each bracket slot system will be followed whenever clinically appropriate. The 0.018-inch bracket slot system archwire sequence will be: 0.016-inch nickel titanium alloy archwire, followed by 0.016 × 0.022-inch nickel titanium alloy archwire followed by 0.016 × 0.022-inch stainless steel archwire. The 0.022-inch bracket slot system sequence will be: 0.016-inch nickel titanium alloy archwire, followed by 0.019 × 0.025-nickel titanium alloy archwire followed by 0.019 × 0.025-inch stainless steel archwire.

**Figure 2:**
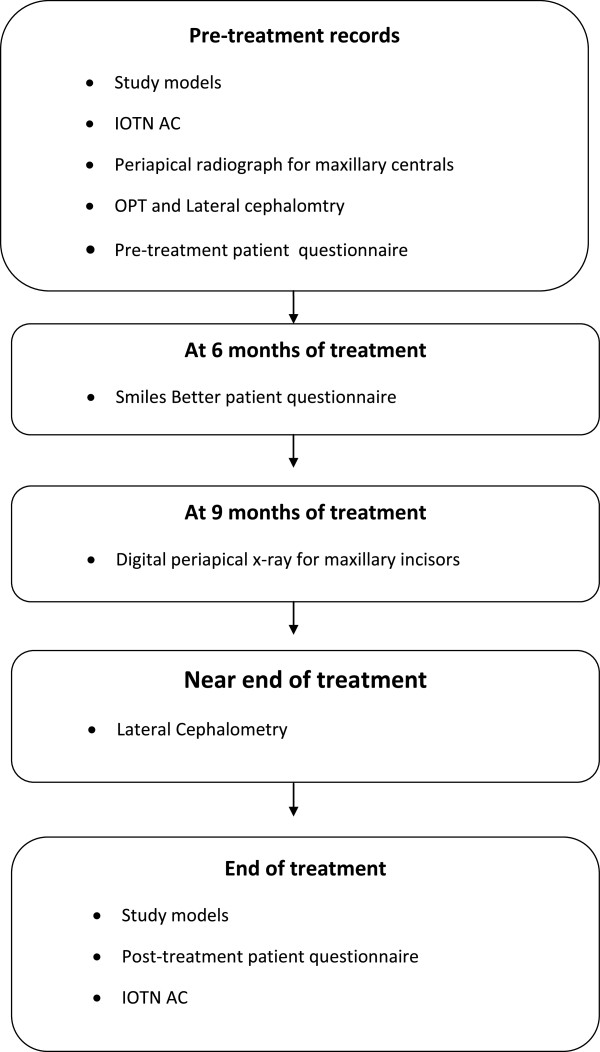
**Trial key steps diagram.** Showing the pre-treatment, mid-treatment and end of treatment records collected. Smile better questionnaire is completed by the study participants with regards to their experience wearing the fixed appliances. Index of Orthodontic Treatment Need Aesthetic Component (IOTN AC), Orthopantomograph (OPT).

A flow chart for the study (Figure 2) showing the key steps will be attached to the participant’s clinical case notes to help remind clinicians to adhere to study protocol. A standardized clinical treatment regime will be used throughout the trial, accepting that clinical circumstances may necessitate deviations from the standard protocol.

### Outcome measures

At the end of the trial, the standardized records will be anonymized before any scoring will be undertaken. Thus the investigator will be blind to the allocation before any observations are recorded in order to eliminate investigator bias.

#### Primary outcome

The primary outcome will be the duration of orthodontic treatment (measured in months) required to finish orthodontic treatment in both the maxillary and mandibular arches, including the number of visits required to complete treatment.

#### Secondary outcomes

The first secondary outcome will be the treatment outcome. This will be measured by evaluating the quality of treatment, the effectiveness of the appliance in delivering torque and the improvement in dental attractiveness. The quality of the treatment result will principally be measured by assessing the occlusal outcome using the Peer Assessment Rating Index (PAR Index) from study models recorded at the start and end of treatment. The torque of the maxillary incisors will be used to evaluate the effectiveness of appliance in delivering effective torque by measuring the angle between the long axis of the central incisors and the maxillary base from lateral cephalograms towards the end of treatment. The improvement in the dental attractiveness of the patient will be calculated using the aesthetic component of the IOTN. This will be evaluated by comparing the patient rating to the attractiveness of his or her teeth before and after treatment.

The second secondary outcome will be the biological side effects of the treatment, as measured by the amount of OIIRR. OIIRR will be established from periapical radiographs recorded at nine months into active treatment. Patients who show evidence of marked OIIRR will be treated in accordance with current recommendations.

The third secondary outcome will be the patient’s perception of wearing the fixed orthodontic appliance and of treatment outcome. This will be recorded using a questionnaire (Smiles Better) at six months after the start of treatment. Patient perception of the of fixed appliance treatment outcome will be measured by comparing patients’ pre-treatment and after-treatment questionnaires.

### Target sample size

The sample size calculation is based on the primary outcome of duration of orthodontic treatment. Using nQuery Advisor 7.0 sample size was calculated to detect a difference in mean treatment duration of three months (clinically important difference). Standard deviation was estimated using the studies of Amditis and Smith [5] and Eberting et al. [12]. A sample size of 92 in each group will have 80% power to detect a difference in means of three months assuming that the common standard deviation is 7.2 using a two group Student’s *t*-test with a 0.05 two-sided significance level. Assuming a 15 to 20% dropout rate, we will recruit a total of 216 patients.

### Participant recruitment

Patients attending the orthodontic department in any of the trial centers for orthodontic treatment who meet the inclusion criteria will be assessed by the clinician planning the orthodontic treatment. The patients will be invited to participate in the study and will be given a participant’s and/or parents’ information sheet to take home with them. Patients will be asked to decide about joining the trial on the next appointment (after at least two weeks).

### Sequence generation and randomization

Simple randomization will be accomplished with no stratification using a restricted (10 number block) random number using http://www.graphpad.com/quickcalcs/randomn2.cfm to ensure equivalence of numbers in each group. The odd numbers will be allocated to group 1 and the even numbers to group 2. In every 10 number block from the random table, the sequence will be checked to ensure the even numbers are equal to the odd numbers. Each number in the random table will be given a study number and assigned into one of the study groups. A table for the allocation of the participants in the study will be composed and kept in a sealed envelope. All the documents used for the randomization and allocation sequence generation will be kept in a box in a locked office away from the clinical environments.

### Participant allocation

Numbered, identical, opaque sealed envelopes will be used for patient allocation in the current trial. The allocation envelope contains the treatment allocation card showing either group 1 or group 2. The allocation envelopes will be kept in a labelled box in an agreed location in the clinical environments. After the clinician obtains informed consent from the patient, a dental nurse will be asked to identify the next allocation envelope in sequence. The allocation will only be revealed at the time of appliance placement. Then the allocation envelope will be opened in front of the participant. Both the participant and the clinician will be informed about the group allocation for the participant.

### Blinding

Due to the nature of the trial it is not possible to blind the clinician to the group once it is allocated. Once the opaque numbered envelope used for random allocation is opened and the clinician and participant know which appliance type (either 0.018-inch slot or 0.022-inch slot) will be used, the appliance will be clearly specified on the pro forma kept within the patient’s case notes. This will allow the clinician to adhere to the recommended standard archwire sequence for each type of appliance.

### Data and records to be collected

The following standardized data will be recorded for the trial participants at three stages of orthodontic treatment. Before start of orthodontic treatment: full orthodontic diagnostic clinical assessment, orthodontically trimmed study models at the start of orthodontic treatment, IOTN (aesthetic component score), long-cone paralleling technique periapical radiographs of maxillary central incisors, lateral cephalometric radiographs recorded before the start of treatment and patient questionnaire (pre-treatment). During orthodontic treatment: treatment duration, number of appointments, dates of key stages in fixed appliance orthodontic treatment, number of fixed appliance components replaced during active treatment, patient questionnaires (Smiles Better) regarding their perception of the impact of treatment (completed by the patient at six months from the start of orthodontic treatment), long-cone paralleling technique periapical radiographs of maxillary incisors recorded at nine months after the start of orthodontic treatment using digital radiography and lateral cephalometric radiograph recorded before the completion of active treatment.

End of orthodontic treatment: orthodontically trimmed study models at the end of treatment, patient questionnaire (post-treatment) and IOTN (aesthetic component score).

### Data management

A data collection sheet designed specifically for the current trial will be used to collect data from all participants’ records including study models, patient questionnaires, case notes and radiographs. The data collection sheet will be coded to allow for the blinding of the investigator to the type of appliance used during data analysis. The data will then be uploaded into a validated excel sheet specially designed for the current trial complying with Good Clinical Practice regulations. At the end of the trial the data will be exported into SPSS (Statistical Package for Social Sciences, Inc., Chicago, Illinois, United States) v.18 for statistical analysis.

### Post-trial care

All study documentation will be retained for at least five years post final data lock. The end of study is defined as the last participant’s last visit. The end of the study will be reported to the Research Ethics Committee (REC) within 90 days, or 15 days if the study is terminated prematurely. A summary report of the study will be provided to the REC within one year of the end of the study.

### Statistical analysis plan (SAP)

#### Descriptive statistics

Descriptive statistics for demographic data (such as age and gender) and baseline measurements will be tabulated by treatment group. Data will be checked for normality of distribution and any evidence of skewness.

#### Statistical analysis

Table 1 shows the planned statistical tests to be used for each variable investigated. The significance level will be set to α = 0.05 (two-tailed).

**Table 1:** **Statistical tests**

Outcome measure		Type of variable	Statistical test ***P***<0.05
**Primary outcome**	Duration of alignment stage for the upper and lower arches	Continuous, assuming normal distribution	One-way ANOVA test
**Secondary outcomes**	Number of visits required	Continuous, assuming normal distribution	One-way ANOVA test
	Occlusal outcome PAR	Continuous, assuming normal distribution	One-way ANOVA test
	Patient perception	Categorical data	Chi-square test
	Severity of OIIRR	Categorical data	Chi-square test or Friedman test

A list of statistical tests that will be used for data analysis depending on the type of data in the current trial. Peer Assessment Rating index (PAR), orthodontically induced inflammatory root resorption (OIIRR), analysis of variance (ANOVA).

### Intention-to-treat and imputation

Participants will be considered part of the intention-to-treat population if they were randomized at the start of the trial. If a participant has missing data for the outcome, then the median value for the treatment group will be imputed. To provide evidence of the robustness of the study results obtained from the above analyses, the same analyses will be repeated on two other data sets; one where imputation is done using the 75th percentile instead of the median, and another using the 25th percentile. These three sets of results will then be compared.

### Stopping rule

During the nine-month periapical radiographic assessment, if severe OIIRR (more than one third of the root Malmgren et al. [13]) is found in the majority of patients in one group, whilst the other group shows minor changes, then a trial monitoring committee will be convened to consider whether the study will be terminated.

### Operator standardization

All patients will be treated in the Dundee Dental Hospital, Perth Royal Infirmary or Springfield Medical Center (Arbroath) by specialist orthodontic staff. Patients will be treated according to the study protocol. No additional appointments will be required due to participation in the study.

### Trial monitoring

The Tayside Medical Science Centre standard operating procedure on adverse event recording will be adhered to for reporting of harms in the current study. Any participant reporting an adverse event would discuss this with the clinic staff or may contact the trial team directly. All adverse events will be recorded from the time participants have the orthodontic appliance bonded until the date of debonding (end of treatment with appliance removal). The trial is considered to be low risk and no adverse events are expected from the fixed orthodontic appliances that are used commonly and safely in most orthodontic units around the world.

### Trial Monitoring Committee

A Trial Monitoring Committee will be established, composed of three researchers (DB, GM and AE). This committee will meet every three months to monitor and discuss data for this trial. If it is found that one intervention particularly is causing significant harm to the dentition then the committee will have to decide to terminate the trial and shift all the study participants to the other intervention.

### Investigator responsibilities

The principal investigators are responsible for the overall conduct of the study at their site and compliance with the protocol and any protocol amendments. Responsibilities may be delegated to an appropriate member of study site staff, which in this case is a senior clinical dental staff member who has been nominated as a co-investigator. Delegated tasks must be documented on a delegation log and signed by all those named on the list.

### Emergency code-breaking procedure

Emergency code-breaking in this randomized control trial is not applicable because the bracket slot size system used for each participant will be clearly detailed within the patient’s clinical notes. The proposed intervention is unlikely to result in significant adverse events. However, should they occur, they will be recorded in keeping with routine clinical practice.

### Ethics and dissemination

#### Ethics approval and consent

The study will be conducted in accordance with the principles of Good Clinical Practice. Approval was obtained from the East of Scotland Ethics Committee (approval number: 09/S1401/56) and National Health Service Tayside Research and Development (approval number: 20092E07). Any changes in research activity, except those necessary to remove an apparent, immediate hazard to the participant, will be reviewed and approved by one of the co-chief investigators. Amendments to the protocol will be submitted in writing to the East of Scotland Ethics Committee and Tayside National Health Service Research and Development.

Informed consent will be taken by the treating clinician (member of the research team) after clarifying any queries asked by the patient and/or the parents. In all trial sites, one of the trial team who is suitably trained will obtain consent from the participant and/or parent(s) or legal guardian(s). All research team clinicians will have had previous Good Clinical Practice training including obtaining informed consent. An independent clinician who is not part of the trial research group agreed to be an independent reference for participants and parents in case there are any further queries regarding the study. His contact information is included in the participant information sheet.

The participant and/or parent(s)/legal guardian(s) in the study will provide written informed consent before any study procedures are carried out and a participant information sheet will be provided to facilitate this process. Those with English as an additional language will be invited to bring an interpreter with them to the subsequent treatment appointment, or to request an NHS interpreter, where this service is available. As part of the consent process participants and/or parent(s)/legal guardian(s) must agree to researchers and regulatory representatives having access to their medical records for monitoring and audit purposes. Participants and/or parent(s)/legal guardian(s) may withdraw their consent to participate at any time during the study. The investigator or delegated member of the trial team and the child should sign and date the assent form whereas the participant (and/or parent(s)/legal guardian(s)) and investigator should sign the informed consent form(s) to confirm that consent has been obtained. The participant should then receive a copy of this document and a copy should be filed in the investigator site file. Patients who refuse to enter the trial will also be recorded. Their demographic and clinical details will be compared at the end of the study to determine that volunteer bias was not present.

#### Withdrawal procedures

Parent(s)/legal guardian(s) will be informed that they have the right to withdraw from the study at any time. The right to refuse to participate without reasons will be respected. After the participant has entered the study the clinician remains free to provide alternative treatment to that specified in the protocol at any stage if he/she feels that it is in the participant’s best interest, but the reasons for doing so will be recorded. In these cases the participants remain within the study for the purposes of follow-up and data analysis. All participants will be free to withdraw at any time from the study without giving reasons and without prejudicing further treatment.

#### Confidentiality

All participants’ records will be identified in a manner designed to maintain participant confidentiality. All records will be kept in a secure storage area with limited access. Clinical information will not be released without the written permission of the participant, except as necessary for monitoring and auditing by the sponsor, its designee, regulatory authorities or the REC. All investigators and study site staff involved with this study will comply with the requirements of the Data Protection Act (UK) 1998 with regard to the collection, storage, processing and disclosure of personal information and will uphold the Act’s core principles. Computers used to gather the data will have limited access via user names and passwords. Published results will not contain any personal data that could allow the identification of participants.

#### Dissemination of results and publication policy

The ownership of the data from the current trial resides with the trial team. On competition of the trial the data will be analyzed and a clinical study report will be prepared. In addition, the results of the trial will be presented at orthodontic national and international conferences and meetings. All patients recruited in the trial will be given a summary of the findings after the final report is finalized.

## Discussion

Orthodontic treatment with fixed appliances usually takes around two years to complete. Therefore this randomized controlled trial presents potential difficulties with participant dropout due to patients moving to other treatment centers and clinicians failing to obtain treatment records at key stages during treatment. Although it is not possible to prevent the former, the latter source of bias will be minimized by regular trial updates and reminders throughout the duration of the trial.

The results from the current study will serve as evidence to guide clinicians in deciding whether the difference in bracket slot size has a significant impact on the effectiveness of orthodontic treatment.

## Trial status

The current trial is open for recruitment since January 2010 and is expected to reach the target enrolment of 216 participants in September 2014.

## Abbreviations

CONSORT: Consolidated standards of reporting trials
IOTN: Index of Orthodontic Treatment Need
NHS: National Health Service
OIIRR: Orthodontically induced inflammatory root resorption
PAR: Peer assessment rating
REC: Research Ethics Committee.
